# Retrospective assessment of rat liver microsomal stability at NCATS: data and QSAR models

**DOI:** 10.1038/s41598-020-77327-0

**Published:** 2020-11-26

**Authors:** Vishal B. Siramshetty, Pranav Shah, Edward Kerns, Kimloan Nguyen, Kyeong Ri Yu, Md Kabir, Jordan Williams, Jorge Neyra, Noel Southall, Ðắc-Trung Nguyễn, Xin Xu

**Affiliations:** 1 National Center for Advancing Translational Sciences (NCATS), 9800 Medical Center Drive, Rockville, MD 20850 USA; 2 NY State Public Health, DOHMH 42-09 28th St, Long Island City, NY 11101 USA; 3 School of Medicine, Virginia Commonwealth University, 1201 E Marshall St, Richmond, VA 23298 USA; 4 The Graduate School of Biomedical Sciences, Icahn School of Medicine at Mount Sinai, 1 Gustave L. Levy Place, New York, 10029 USA

**Keywords:** Computational models, Cheminformatics, Small molecules, High-throughput screening, Mass spectrometry

## Abstract

Hepatic metabolic stability is a key pharmacokinetic parameter in drug discovery. Metabolic stability is usually assessed in microsomal fractions and only the best compounds progress in the drug discovery process. A high-throughput single time point substrate depletion assay in rat liver microsomes (RLM) is employed at the National Center for Advancing Translational Sciences. Between 2012 and 2020, RLM stability data was generated for ~ 24,000 compounds from more than 250 projects that cover a wide range of pharmacological targets and cellular pathways. Although a crucial endpoint, little or no data exists in the public domain. In this study, computational models were developed for predicting RLM stability using different machine learning methods. In addition, a retrospective time-split validation was performed, and local models were built for projects that performed poorly with global models. Further analysis revealed inherent medicinal chemistry knowledge potentially useful to chemists in the pursuit of synthesizing metabolically stable compounds. In addition, we deposited experimental data for ~ 2500 compounds in the PubChem bioassay database (AID: 1508591). The global prediction models are made publicly accessible (https://opendata.ncats.nih.gov/adme). This is to the best of our knowledge, the first publicly available RLM prediction model built using high-quality data generated at a single laboratory.

## Introduction

Hepatic metabolic stability is a key parameter in drug discovery because it can prevent a drug from attaining sufficient in vivo exposure, producing short half-lives, poor oral bioavailability and low plasma concentrations. It is essential to identify metabolic liabilities early in drug discovery so they can be addressed during lead optimization. Metabolic stability is typically first measured in vitro using liver microsomes and data from this assay is used to guide structural modifications to improve stability or select the best compounds for in vivo pharmacokinetic (PK) and efficacy testing. Liver microsomes are enriched with cytochrome P (CYP) 450 enzymes, localized in the endoplasmic reticulum membrane, which are responsible for the metabolism of the majority (70–80%) of clinically approved drugs^1,2^. The National Center for Advancing Translational Sciences (NCATS) determines metabolic stability initially with a high-throughput, substrate-depletion method (i.e. the in vitro half-life (t_1/2_) approach) in rat liver microsomes (RLM).

The rationale behind using RLM as the matrix for initial screening is twofold: (1) rat is a key species for both initial in vivo PK studies^3^ as well as later efficacy and toxicology studies; and (2) rat PK data predicts human PK data reasonably well with single-species allometric scaling^4,5^. In vitro RLM data is routinely used to predict in vivo clearance in rats. This in vitro data is also useful to set up in vitro*-*in vivo correlations which can provide confidence in extrapolating in vitro data to in vivo clearance for other species, including humans. Our assay (96-well format; adaptable to 384-well format) consists of a validated automated liquid handling process for incubation and sample clean-up and a comprehensive LC/MS method to determine the percent of remaining parent compound at the end of incubation.

Although experimental metabolic stability data provides useful information, a prediction model will be extremely useful to help design new compounds and prioritize which compounds to synthesize first. Quantitative structure activity relationship (QSAR) models for predicting RLM stability exist in the literature^6,7^, however, these are proprietary. Surprisingly, little or no literature exists that describes the use of modern deep learning approaches to develop QSAR models for RLM stability.

NCATS has generated RLM stability data for nearly ~ 24,000 compounds from more than 250 therapeutic projects since 2012. The primary goal of this study is to build QSAR models using these structurally diverse compounds for predicting hepatic metabolic stability. Furthermore, a retrospective analysis of the historical data and prediction models was performed to identify best modeling strategies for ongoing and future projects at NCATS. Substructure analysis over a diverse chemical space revealed transformation rules that can potentially be useful to chemists in addressing problems with highly unstable compounds. Data for ~ 2500 compounds is made publicly available to encourage the community to develop and validate in silico models using a high-integrity reference data set. All prediction models were developed using open-source software and are made available to the scientific community.

## Material and Methods

### Materials

Dimethyl sulfoxide (DMSO, UPLC/MS grade), albendazole, buspirone, propranolol, loperamide, diclofenac, carbamazepine, antipyrine, potassium phosphate monobasic, and potassium phosphate dibasic were purchased from Sigma-Aldrich (St. Louis, MO). Acetonitrile (ACN, UPLC/MS grade) was purchased from Fisher Scientific (Hampton, NH). Gentest rat (Sprague–Dawley) liver microsomes (male, pooled, 20 mg/mL, Catalog #: 452501), Gentest NADPH Regenerating Solution A (Catalog #: 451220) and B (Catalog #: 451200), Axygen reservoirs (low-profile, Catalog #:RES-SW384-LP; high-profile, Catalog #: RES-SW384-HP) for holding reagents, and CoStar assay block plates (Catalog #: 3959) for microsomal incubation were purchased from Corning Inc. (Corning, NY). LC–MS/MS analysis plates (Catalog #: 186002643) were purchased from Waters Inc. (Milford, MA).

### Microsomal stability assay

Microsomal stability of the test articles was determined in a high-throughput format using the substrate depletion method^8^. It has been previously shown that in vitro intrinsic clearance does not vary significantly between the three most used rat strains^9^. Sprague-Dawley was chosen because it is the most common strain used in drug discovery. Experiments were performed using a Freedom Evo 200 automated platform with a 96-channel (MCA96) head with EVOware software (version 3.2) (Tecan Inc., Männedorf, Switzerland). The system also includes an Inheco heating block and cooling block (Inheco, Munich, Germany). Six standard controls were tested in each run: buspirone, propranolol, diclofenac, loperamide, carbamazepine and antipyrine. The assay incubation system consisted of 0.5 mg/mL of rat microsomal protein, 1.0 μM drug concentration, and NADPH regeneration system (containing 0.650 mM NADP + , 1.65 mM glucose 6-phosphate, 1.65 mM MgCl_2_, and 0.2 unit/mL G6PDH) in 100 mM phosphate buffer at pH 7.4. The incubation was carried out at 37 °C for 15 min. The reaction was quenched by adding 555 μL of acetonitrile containing 0.28 μM albendazole, an internal standard. After a 20-min centrifugation at 3000 rpm at 4 °C, 30 μL of the supernatant was transferred to an analysis plate and was diluted fivefold using 1:2 v/v acetonitrile/water. Sample quantification and analysis were performed using a previously described method^10^ with minor modifications. t_1/2_ values were capped at 30 min since for a 15-min assay, the data cannot be extrapolated beyond 30 min^8^.

### RLM stability data set

A 15-min single-point assay allows measurement of highly unstable compounds (t_1/2_ values from 1 to 5 min) while providing an upper t_1/2_ limit of 30 min. This is a good working range for drug discovery, where the primary concern is identifying highly unstable compounds^11^. Concerns decrease at t_1/2_ greater than 30 min and hence, compounds were classified as unstable (t_1/2_ < 30 min), or stable (t_1/2_ > 30 min)^11–13^. Compounds that have extremely short t_1/2_ tend to have high clearance and low in vivo oral bioavailability. Improving the t_1/2_ beyond 30 min has generally been shown to decrease clearance and increase bioavailability^14,15^. We employ this cutoff internally at NCATS and several projects have benefitted from this approach^16,17^. The raw data set was preprocessed to generate training and test data for the purpose of building and validating prediction models. Compound structures were normalized following best practices recommended in the literature^18^. LyChI identifiers (https://github.com/ncats/lychi) were generated for all standardized structures to identify unique compounds. Further, compounds with conflicting experimental results were omitted. Finally, the processed data set comprised a total of 20,216 (Unstable: 11,534; Stable: 8682) compounds. Detailed steps involved in preprocessing the raw data set are provided in the supplementary information (Table S1).

### Cross validation strategies

The preprocessed RLM data set was initially partitioned into training and test sets using ‘*train_test_split*’ module from Scikit-learn^19^, a Python library for machine learning. The split was performed a total of five times with the *shuffle* parameter set to *True*, following a five-fold cross-validation (5-CV). In each split, 80% of the shuffled data set was assigned to the training set and the remaining 20% to the test set. The training sets were used to build models and the test sets to validate them. This refers to the first cross-validation scheme employed in this study.

**Figure 1:**
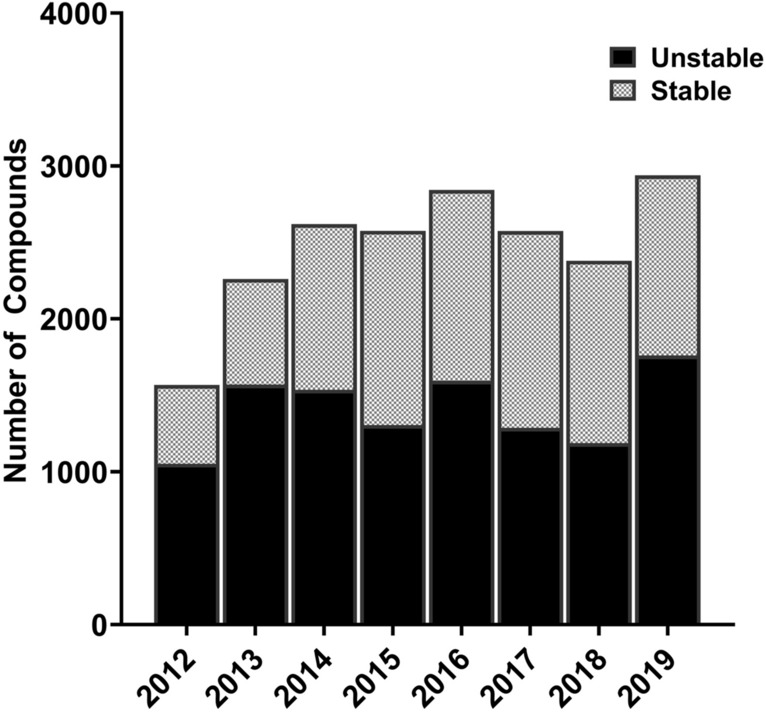
Time-split distribution of RLM stability data (2012 to 2019).

In a typical drug discovery set up, compounds are tested in an appropriate assay and models are usually built using this data around the same time. These models are then used to predict the properties of compounds that are not yet synthesized. However, these compounds may or may not fall within the applicability domain of the model. In this context, ‘*time-split*’ was previously proposed as a cross-validation strategy that closely simulates a prospective validation scenario^20^. Briefly, one would generate a model based on data available at a certain time point and test the model on data generated later. It was previously demonstrated on large data sets from Merck that the performance of models based on time-split cross-validation more closely resembles prospective validation than *random-split* cross-validation and others^21^. The availability of chronological RLM stability data from 2012 to 2019 enabled us to perform a *time-split* cross-validation in this study. Therefore, we used data from each year to build models and validated them on the data that were generated in the next year. For each test year, all data available from the previous years combined is considered as the training set. Thus, a total of eight *time-split* models were built for seven consecutive years (2012 to 2019) in a cumulative fashion (i.e., for 2019, all data generated until 2018 was used to build a model and tested on data from 2019). Distribution of data across the two stability classes over different years is provided in Fig. 1.

### Modeling methods

#### Random Forest

Random Forest (RF)^22^ is an ensemble of decision trees that are fitted on various subsamples of the data and uses averaging to restrict overfitting and improve prediction accuracy. The ‘*RandomForestClassifier’* method from Scikit-learn was used to build models. The number of estimators per model was set to 100 and the random state was set to an integer. The rest of the parameters were set to default.

#### Deep neural networks

Artificial neural networks (ANN) have been applied to a wide range of QSAR tasks^23,24^. More recently, the ANNs have evolved into deep neural networks (DNN)^25^. Unlike an ANN, a DNN consists of multiple fully connected layers with two or more hidden layers between the input and output layers. In a feedforward neural network (referred to simply as DNN in the rest of the study), the information passed through the input layer flows in forward direction through the hidden layers to the output layer. The DNN models were implemented in Keras (https://keras.io) using the TensorFlow (www.tensorflow.org) backend. The number of hidden layers was adjusted based on the size of the input descriptor matrix. More details on model parameters are presented in supplementary information (Table S2).

#### Graph convolutional neural networks

Molecular graphs provide a natural way of describing chemical structures: nodes represent atoms and edges represent bonds. The recently emerged graph convolutional neural networks (GCNN)^26,27^ that can be operated on molecular graphs have been used extensively for molecular property predictions^28,29^. The message passing variant of GCNN, as implemented in ChemProp^28^, was employed in this study adhering to the default parameters. The graph features are internally computed when chemical structures and associated labels are provided as input.

#### Recurrent neural networks

A recurrent neural network (RNN) is a type of neural network that can store information within the network. RNNs are able to learn sequence data such as natural language. Long-Short-Term-Memory (LSTM) networks are a type of RNN that use special units in addition to standard units to store information for longer periods of time^30^. Former studies reported the use of LSTMs to learn directly from linear molecular representations such as simplified molecular-input line-entry system (SMILES)^31–34^. The LSTM networks built in this study were fed with canonical SMILES representations that are first encoded into one-hot vectors and then passed to the computing cell which performs as many computations as the length of the input SMILES in a loop. At each step, one character of SMILES is taken as input and the computed activation value is passed to the next step which takes the next character as input. In this way, the information from previous characters is persisted while the next characters are being processed. Finally, the network produces a prediction probability between 0 and 1.

### Molecular descriptors

Four different molecular descriptors were used in this study. To represent the compounds in the physicochemical property space, RDKit descriptors were calculated using RDKit Descriptor calculation node in KNIME^35^. Fingerprints on the other hand are bit vectors that encode chemical structures in their two-dimensional (2D) space. In this study, Morgan fingerprints (an extended-connectivity fingerprint)^36^ containing 1024 bits with a radius of 2 were calculated using RDKit Fingerprint node in KNIME. Similarly, Avalon fingerprints^37^ are hashed fingerprints that enumerate paths and feature classes. Avalon fingerprints of length 1024 bits were calculated using the same KNIME node. Both RF and DNN models use the RDKit descriptors and the two fingerprints for model development. GCNNs directly use the 2D molecular graphs to generate graph featurization for every compound. The LSTM networks directly operate on SMILES notations.

### Validation metrics

The performance of the models was assessed using different statistical measures. A receiver operating characteristic curve plots the true positive rate against the false positive rate and thus provides an estimate of the performance of a binary classifier. The area under the receiver operating characteristic curve (AUC-ROC) was calculated for this purpose. The sensitivity (or the true positive rate) of a model is the proportion of unstable compounds correctly predicted as unstable. Specificity (or the true negative rate) is the proportion of stable compounds correctly predicted as stable. Balanced accuracy (BACC)^38^ is an average of the proportions correctly predicted for each class (i.e., Sensitivity and Specificity). Cohen’s *Kappa* is another performance metric used to evaluate the models in this study. It was originally proposed to measure the agreement between two judges based on accuracy adjusted for a chance agreement. In the sense of classification, it is a measure of the agreement between the actual classes and the classes predicted by a classifier^39^.

$$Sensitivity (Sens)=\frac{\mathrm{TP}}{(\mathrm{TP}+\mathrm{FN})}$$

$$Specificity (Spec)=\frac{\mathrm{TN}}{(\mathrm{FP}+\mathrm{TN})}$$

$$Balanced \, accuracy (BACC)=\frac{Sensitivity+Specificity}{2}$$

$$Kappa=\frac{{p}_{a}- {p}_{\in } }{1- {p}_{\in }}$$

Here, TP = number of true positives; FN = number of false negatives, TN = number of true negatives, and FP = number of false positives. In the case of *Kappa*, ${p}_{a}$ is the proportion of observations in agreement and ${p}_{\in }$ is the proportion in agreement due to chance.

## Results

### Metabolic stability assay performance

Six control compounds were run routinely in each assay plate. The assay reproducibility data for these compounds across 600 experiments, spanning a timeline of eight years are presented in Table 1. The minimum significant ratio (MSR)^40^ for all control compounds was around 2.0, which demonstrates excellent assay reproducibility over a wide range of metabolic stabilities. Since the t_1/2_ data cannot be extrapolated beyond 30 min^8^, the standard deviation (S.D) and MSR values were not calculated for the highly stable control compounds. The inter-assay reproducibility (% CV) for most non-control compounds with at least 4 replicates was found to be < 20% indicating the robustness of our assay (Supplementary information Table S3).

**Table 1:** Reproducibility data for control compounds. Mean and S.D of the t_1/2_ values were calculated for exemplary controls across 600 plates.

Compound	t_1/2_ (min)	MSR $({10}^{2\sqrt{2}\boldsymbol{*}{\varvec{S}}.{\varvec{D}}})$
Buspirone	3.8 ± 1.1	2.1
Propranolol	1.4 ± 0.3	1.7
Diclofenac	11.4 ± 2.6	1.8
Loperamide	8.9 ± 2.4	1.9
Antipyrine	> 30	N/A
Carbamazepine	> 30	N/A

### Chemical space and data distribution

**Figure 2:**
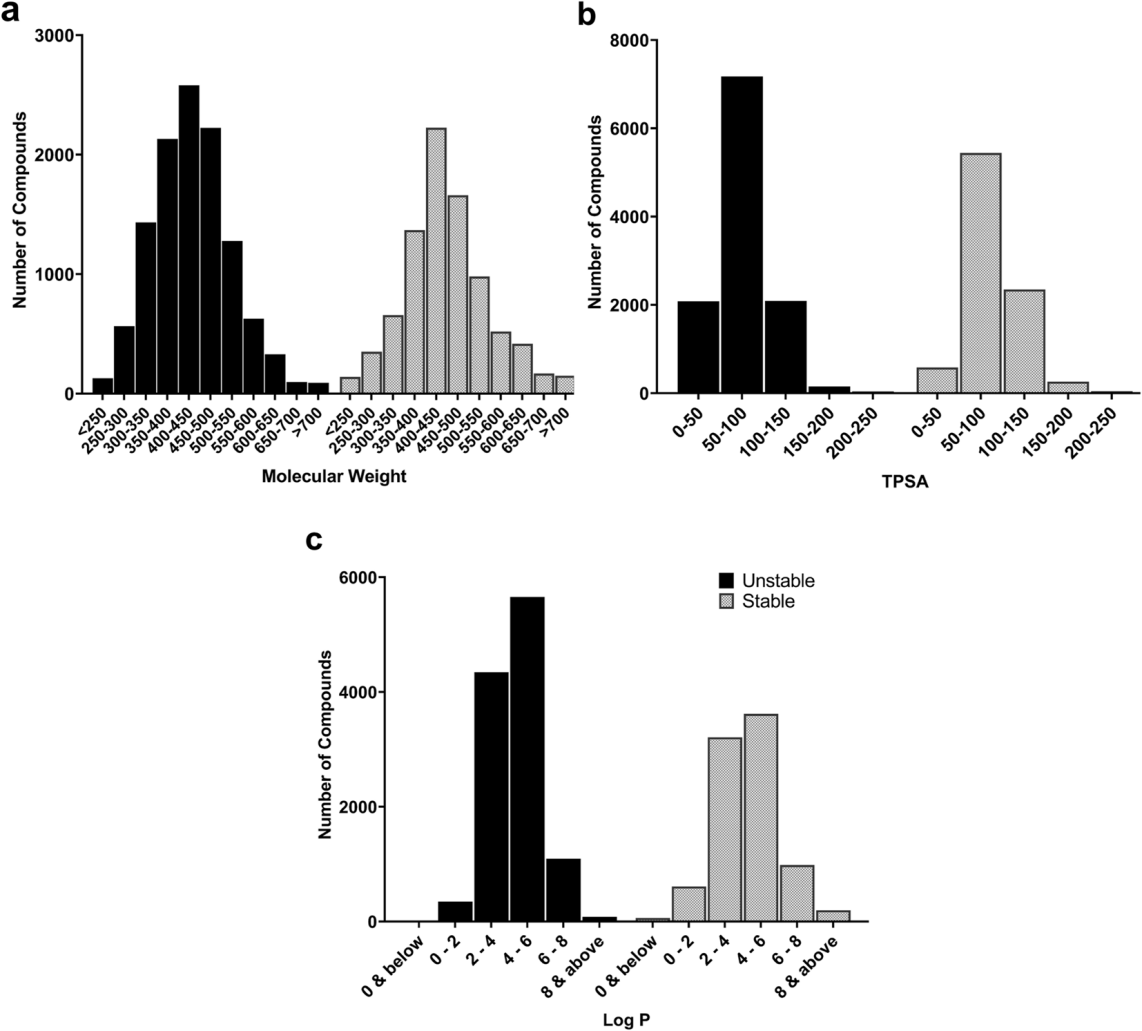
Distributions of the data based on: (**a**) Molecular weight, (**b**) TPSA, (**c**) and Log P.

To understand the data at hand, distributions based on in vitro t_1/2_ and different molecular properties (log P, total polar surface area and molecular weight) were examined. The post-processed data set is slightly skewed towards unstable compounds (~ 58%) compared to stable compounds (~ 42%). Further, nearly 40% of the majority class compounds were found to be extremely unstable (t_1/2_ ≤ 5 min) (Data not shown). The time-split nature provides an alternative view of the data (Fig. 1). A majority of compounds belong in the 300–500 molecular weight range (Fig. 2a), have total polar surface area (TPSA) below 100 (Fig. 2b) and have log P values in the range of 2.5 to 7.5 (Fig. 2c). No significant differences were found between the classes in terms of their distribution based on different molecular properties.

**Figure 3:**
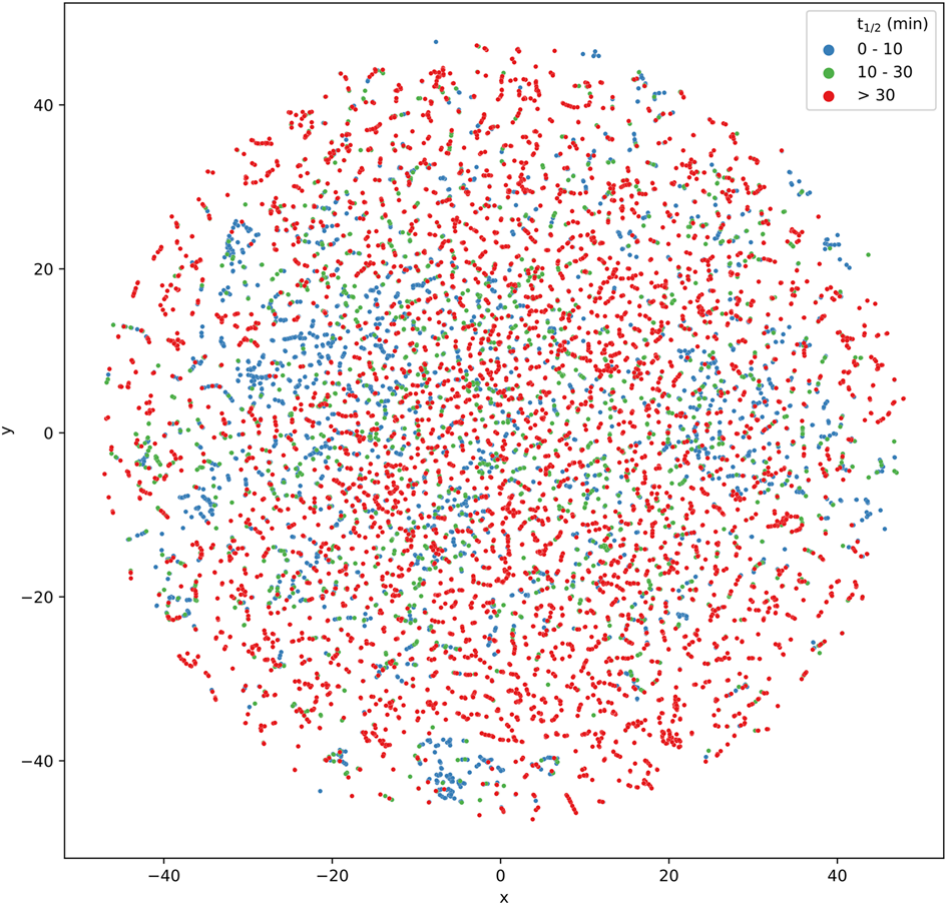
Visualization of the chemical space of RLM stability data set. The axes labels *x* and *y* indicate the first two dimensions of the t-SNE embedding.

To examine the chemical space coverage of the data set, the compounds were projected into a low-dimensional space using the t-distributed Stochastic Neighbor Embedding (t-SNE) method^41^. RDKit descriptors were employed for generating the two-dimensional (2D) chemical space representation (Fig. 3) considering the computational costs involved in processing a large number of descriptors for more than 20,000 compounds. Each point in the 2D space represents a chemical compound and the color denotes the t_1/2_ group as shown in the color palette. The algorithm tries to covert similarities between the compounds to joint probabilities and minimize the Kullback–Leibler divergence^42^ between the joint probabilities of the high-dimensional data and the low-dimensional embedding. Since the number of descriptors was not too high, dimension reduction was not applied prior to projection of compounds into the 2D space.

**Figure 4:**
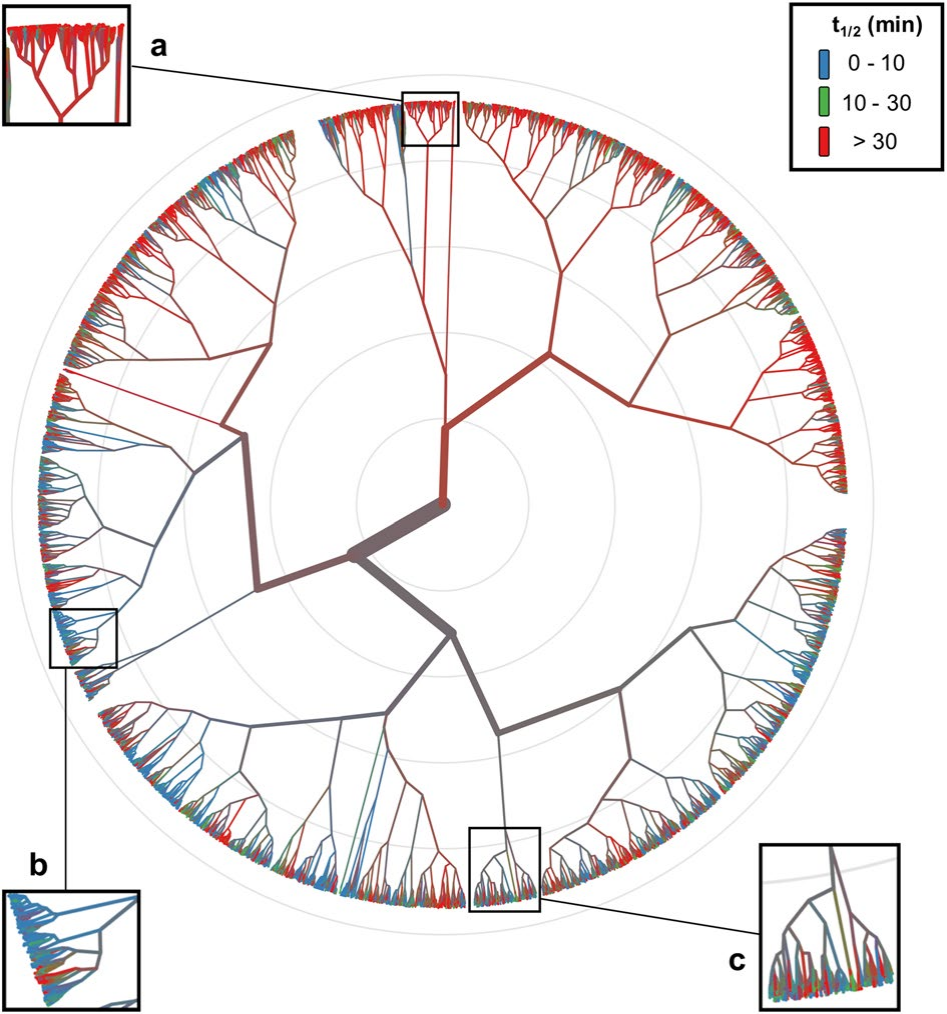
Hierarchical clustering of the RLM stability data set. Exemplary regions that represent: (**a**) abundance of highly stable compounds; (**b**) abundance of highly unstable compounds; and (**c**) a mixture of compounds belonging to different t_1/2_ groups; are highlighted.

Clearly, no simple separation could be detected between the stable compounds and the different subgroups of unstable compounds although compounds from the former group are dominant in number. A complex separation between the groups and presence of several small clusters comprising compounds from all three categories suggest that there might exist several compound pairs with a minor structural change leading to large differences in t_1/2_. This was confirmed by performing a hierarchical clustering of the compounds. We used the same three t_1/2_ groups that were used in visualizing the chemical space. Hierarchical clustering was performed in R 3.6.3 based on a Euclidean distance matrix, calculated using non-correlating RDKit descriptors (84 out of 119), and Ward linkage method^43,44^. The output is represented as a circular dendrogram (Fig. 4) generated using the R package ‘polarClust’ (https://github.com/backlin/polarClust), while retaining the color palette used for chemical space visualization. Rand Index^45^ was calculated in order to quantitatively assess the quality of clustering with respect to the original class labels. While the Rand Index was found to be 0.68 when all three t_1/2_ groups were clustered, the value increased to 0.92 when only the first two groups were considered (i.e., excluding compounds with t_1/2_ > 30 min). This could be explained by the presence of many compounds whose actual t_1/2_ values could not be exactly determined. Overall, the clustering results were mixed because there appeared different types of clusters: some with an overrepresentation of stable compounds (Fig. 4a); some with large number of highly unstable compounds (Fig. 4b) and some compounds from all three groups (Fig. 4c).

### Cross-validation results

**Figure 5:**
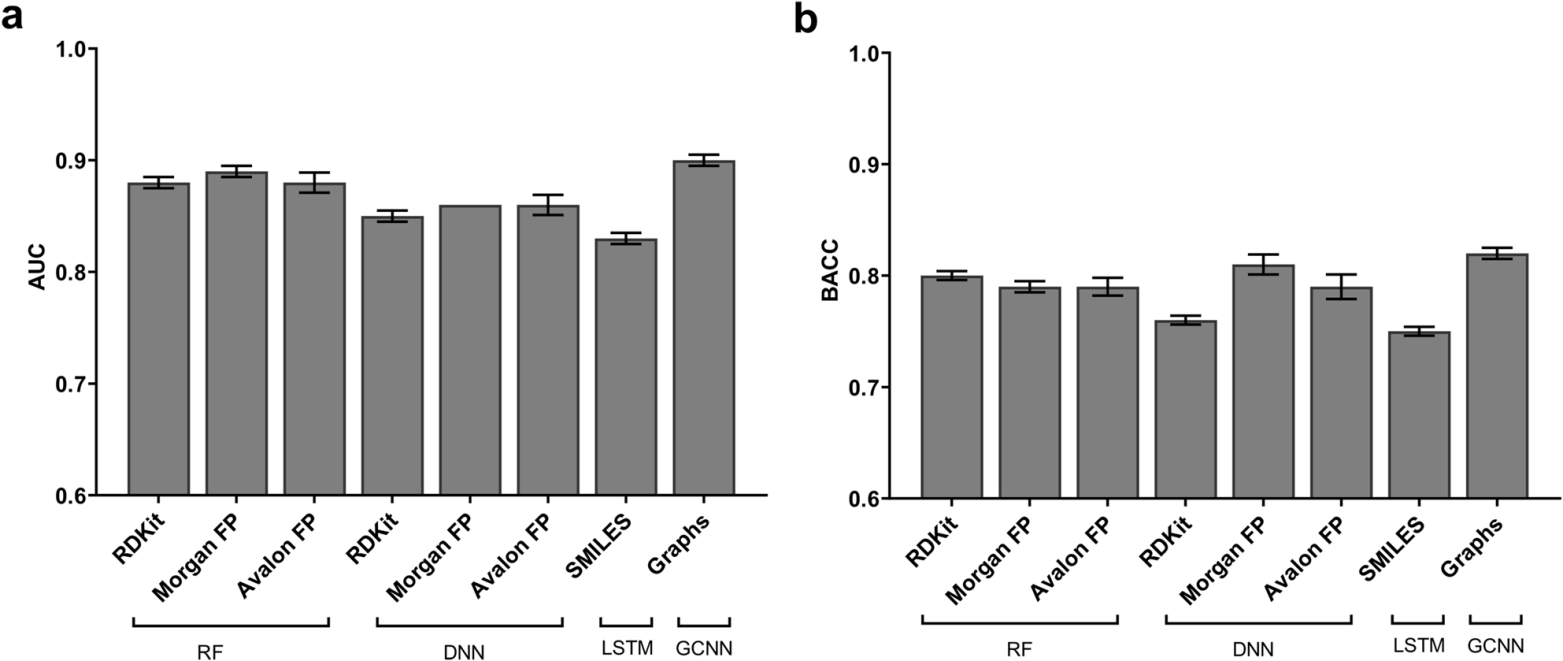
Results of the eight models evaluated in five-fold cross-validation. (**a**) Performance measured as AUC. (**b**) Performance measured as BACC. The standard deviation of the average over five folds is represented as an error bar for each model.

Since our initial idea was to build models using the complete training data and perform five-fold cross-validation, we first present results for all methods and descriptor types. A total of eight models were evaluated at this stage and there were only modest differences between them (Fig. 5; supplementary information, Table S4). In the case of RF and DNN models, no significant differences were found between the performances of RDKit descriptors and two fingerprints (Morgan and Avalon). RDKit descriptors seem to provide the best balance between model sensitivity and specificity in the case of RF. Similarly, Morgan fingerprints provided the highest balanced accuracy for the DNN models. The LSTM model provided the worst performance of all models evaluated at this stage. On the other hand, the GCNN model provided the highest AUC and BACC. Although none of the models unequivocally outperformed the remaining models, the GCNN model provided the highest Cohen’s *Kappa* (0.64).

Next, we performed time-split cross-validation to evaluate the models prospectively. Since the number of compounds generated in 2013 is higher than the total number of compounds available from the year 2012, we decided to begin time-split validation when at least 5000 compounds were available from the previous years. Thus, the data generated from years 2012, 2013 and 2014 (6448 compounds) were used to build a model that is validated on the data generated in the year 2015 (2576 compounds), and so on. In each case, the model is named after the year from which the test data was employed (e.g., Model 2015 is based on training data generated until 2014 and validated on test data from 2015). Thus, a series of five models (Model 2015 to Model 2019) were built and evaluated. Only the best performing descriptors were employed for RF (RDKit descriptors) and DNN (Morgan fingerprint).

**Figure 6:**
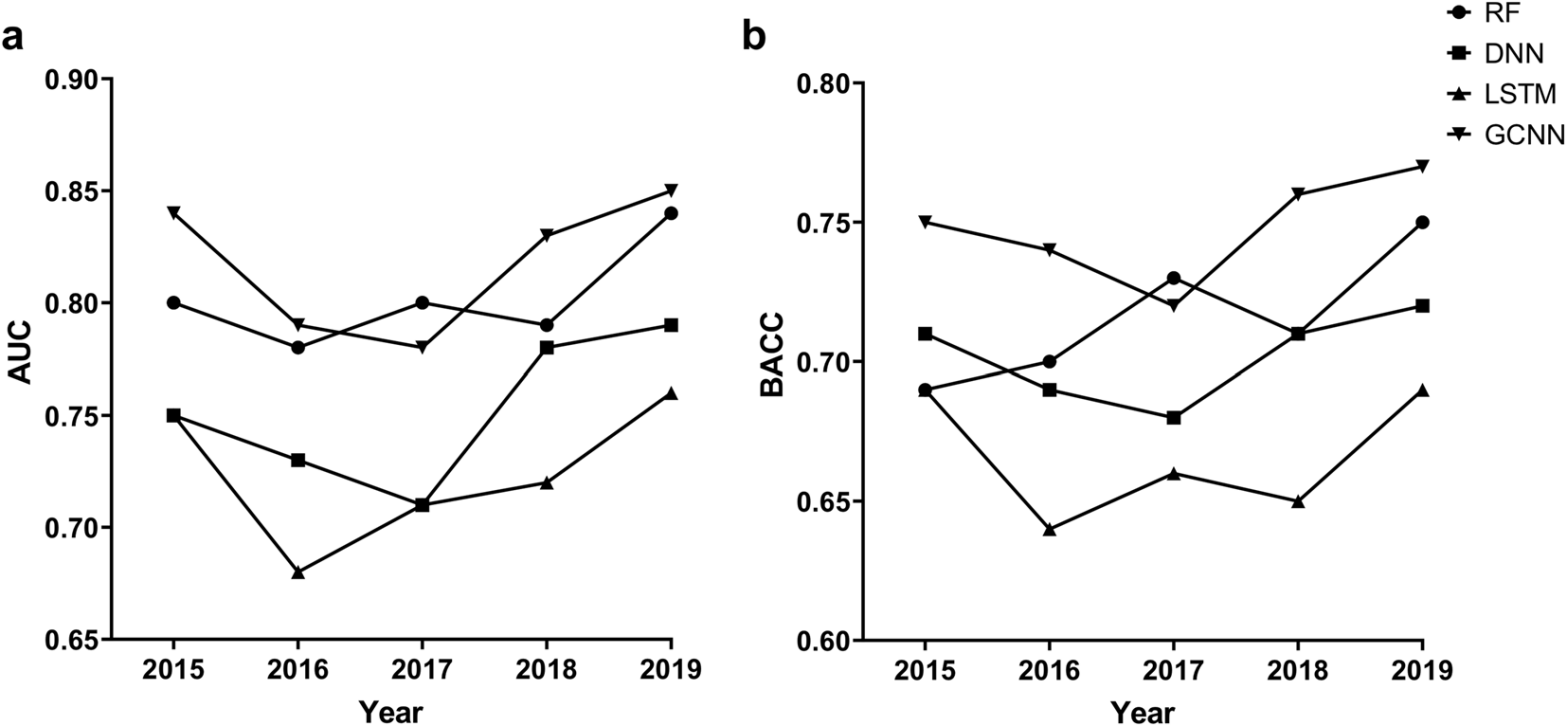
Time-split validation results for the four modeling methods. (**a**) Performance measured as AUC. (**b**) Performance measured as BACC.

Time-split validation results are presented in Fig. 6 and supplementary information (Table S5). GCNN remained the best performing method in all years except 2017 where RF performed slightly better, both in terms of AUC (Fig. 6a) and BACC (Fig. 6b). RF and DNN followed GCNN in terms of performance. Across all years, the LSTM method consistently provided the worst performance. While none of these methods showed a constant rise or decline over the five years, GCNN provided the most consistent performance.

### Global models versus local models

**Figure 7:**
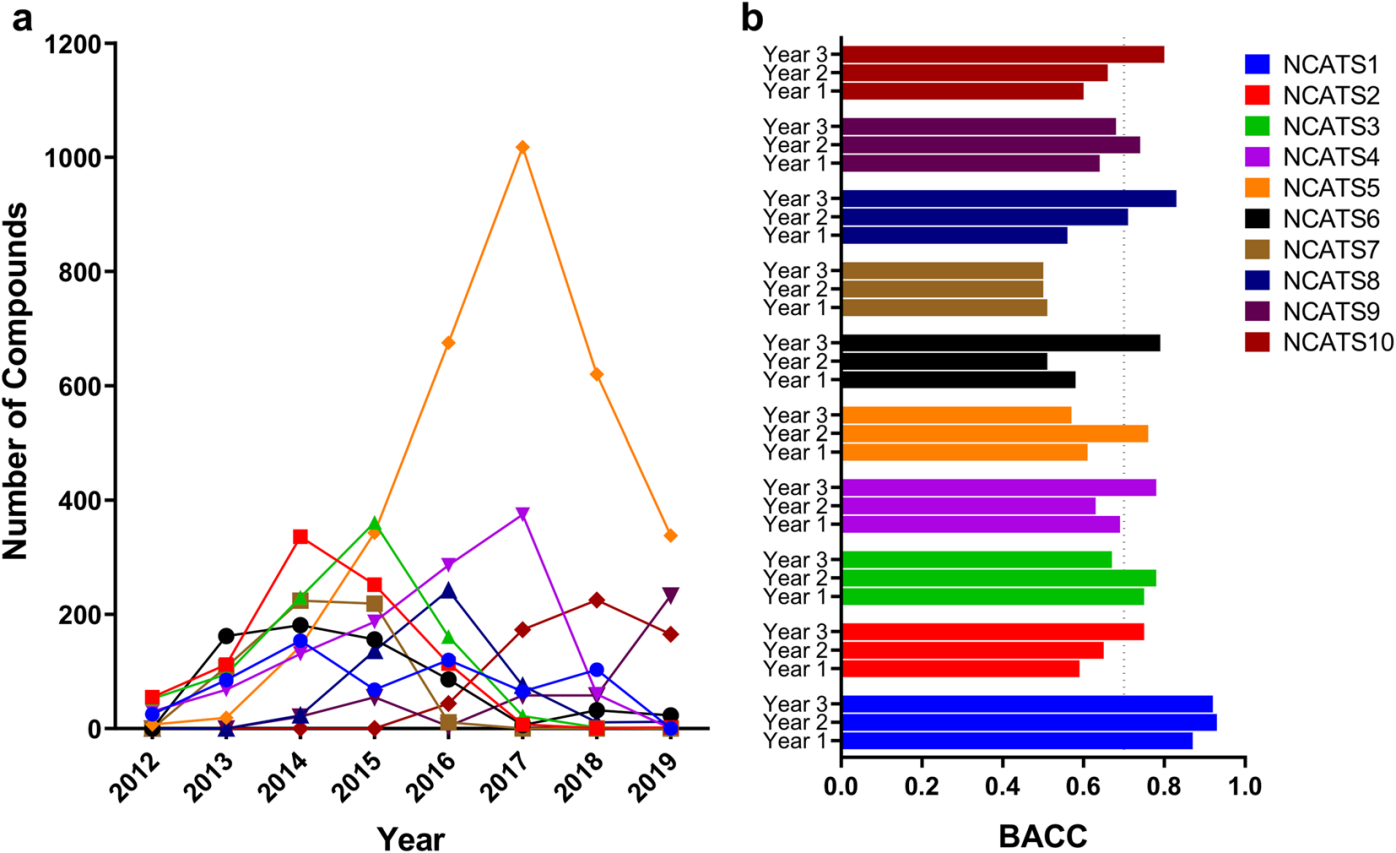
Top 10 NCATS projects chosen for retrospective analysis. (**a**) Distribution of compounds for all 10 projects across multiple years. (**b**) Performance of global models on data from the 10 projects in three consecutive years (Year 1, Year 2 and Year 3). The dotted line represents the BACC threshold of 0.7.

Ten medicinal chemistry projects with the highest number of compounds measured in the RLM assay were examined in detail (Fig. 7). The names of the projects were anonymized (NCATS1 to NCATS10) for the purpose of this study.

For each project, data from the three most recent years was considered, provided there were at least 50 compounds measured in each year (NCATS1, NCATS4, NCATS5, NCATS9 and NCATS10). When recent data were not available, we considered those three consecutive years between 2012 and 2019 where the highest number of compounds had been tested for a project (NCATS2, NCATS3, NCATS6, NCATS7 and NCATS8). The time-split models that were previously built for individual years were used to predict these test sets. It must be noted that the actual number of compounds per year might vary slightly from those reported in Fig. 7a due to chemical structure standardization and other preprocessing steps. The performance of models (in terms of BACC) for these projects are presented in Fig. 7b.

The BACC values for every project, except NCATS7, reached values greater than 0.75 for at least one of the three years and for some projects, even higher BACC values were achieved. The performance for most projects increased with time, most likely due to presence of similar compounds in the training set. Next, we built local models for projects that fared poorly (NCATS5 and NCATS7) with the global models. The BACC improved drastically when we employed the local model approach for the poorly performing projects. Particularly, in the case of NCATS5, the performance increased consistently (Table 2) with a local model in contrast to the global model where there was a sharp decline in year three. This could be due to introduction of novel chemotypes that were not covered by the training data accumulated at the end of second year. We also noticed that the best performing local model for NCATS5 was based on DNN using Morgan fingerprints and not GCNN which performed the best in our global models. Overall, the global models did not perform superiorly for every project, suggesting that local models should be developed for projects that may not benefit from the former approach. Thus, no method demonstrated a clear advantage and determining the best approach may not be straightforward.

**Table 2:** Performance of the global and the local models generated for the project NCATS5.

Year	# Compounds in Training Set	# Compounds in Test Set	BACC (global)	BACC (local)
2017	1188	1018	0.61	0.60
2018	2206	620	0.76	0.71
2019	2862	338	0.57	0.75

### Matched molecular pair analysis

Based on the consistent findings from both chemical space distribution and hierarchical clustering, we sought to perform detailed analysis of the data set to explore and identify medicinal chemistry rules that could potentially serve as guidance for the chemists in tackling metabolic stability issues during lead optimization. Availability of a large number of compounds in the data set facilitated a large-scale matched molecular pair analysis (MMPA). A matched molecular pair can be defined as a pair of compounds that differ at only one site through a minor structural transformation that leads to a difference in an associated property value^46,47^. In a recent analysis^48^, we showed the utility of MMPA in analyzing human and mouse cytosol stability data. The extracted rules were experimentally validated and the extent of their applicability on newly generated data was demonstrated. Herein, well-defined structural changes that lead to an increase in t_1/2_ can be considered potentially useful to address microsomal metabolism liabilities of small molecules. The complete matched pairs analysis was performed in KNIME using the Vernalis and RDKit nodes following the fragmentation scheme originally proposed by Hussain and Rea^49^. In order to prospectively validate the transformations, the data set was randomly divided into two parts at a ratio of 80:20. The bigger partition was used to generate the rules that were supposed to be validated on the smaller partition. However, an upper cap of 60 atoms per molecule was applied to the 80% partition which resulted in ~ 11,500 compounds. The rules were generated from this subset and validated on the remaining ~ 8,500 compounds.

67,000 unique transformations (only left to right transforms were considered) were identified after grouping the obtained matched pairs. Filtering them further by including only those with a frequency of at least 15 instances in the training set resulted in a total of 397 matched pairs. Only those pairs that resulted in an increase in t_1/2_ by at least 10 min were retained for further inspection. A total of 18 such transformations were identified that were present in 349 compound pairs within the training set. These transformations were applied to the remaining compounds using RDKit’s ‘Apply Transforms’ node which resulted in ~ 138,000 new compound pairs. The results were further filtered to retain only those pairs where the molecules on right side (generated after applying transformation) are already present in the RLM data set. This facilitates quantitative validation of the rules. A total of 286 such compound pairs, representing all 18 rules, could be identified through LyChI lookup. The transformations and all related statistics from both training and test data used for MMPA, are presented in Table 3.

**Table 3:** Detailed statistics on the 18 transformations selected from the MMPA.

Fragment (L)	Fragment (R)	Training				Test			
		Compound Pairs	Average t_1/2_ (min)			Compound Pairs	Average t_1/2_ (min)		
		Total (+ ve/und/−ve)	L	R	R/L	Total (+ ve/und/−ve)	L	R	R/L
		25 (23/1/1)	6	29	10.8	68 (57/8/3)	9.4	28.4	7.7
		41 (38/3/0)	9.5	29.7	9.8	49 (41/7/1)	9.3	29.0	8.6
		19 (15/2/2)	11.8	23.8	6.5	9 (6/1/2)	11.2	13.5	1.5
		15 (10/5/0)	13	24.3	6.5	5 (3/0/2)	5.2	7.9	1.3
		17 (9/7/1)	16.3	27.8	5.3	6 (5/1/0)	9.5	21.5	6.3
		15 (9/4/2)	13.9	24.3	5.1	3 (2/0/1)	11.0	12.9	5.5
		17 (15/1/1)	11.8	25.3	4.8	7 (6/1/0)	8.2	17.9	2.8
		15 (9/6/0)	16.1	28	4.3	10 (4/2/4)	13.5	16.3	3.0
		15 (9/6/0)	17	27.8	4.3	8 (5/0/3)	9.9	17.2	2.0
		15 (10/4/1)	13.5	24.4	3.8	5 (4/0/1)	11.3	16.0	2.2
		18 (14/3/1)	12.8	23.1	3.8	10 (9/0/1)	7.4	16.6	2.2
		21 (19/2/0)	7.7	17.9	3.6	23 (16/0/7)	6.6	12.3	3.6
		23 (14/8/1)	13.3	23.4	3.5	43 (26/5/12)	11.1	18.3	4.0
		25 (20/4/1)	12.1	27.3	3.5	11 (7/3/1)	15.7	25.2	2.2
		21 (10/11/0)	19.3	29.4	3.4	10 (6/4/0)	16.8	29.5	3.4
		17 (14/3/0)	15.3	27.2	2.6	13 (7/4/2)	11.8	21.1	4.5
		15 (15/0/0)	15.4	29.8	2.3	3 (3/0/0)	15.3	30.0	2.0
		15 (15/0/0)	15.4	29.8	2.3	3 (3/0/0)	15.3	30.0	2.0

The compound pairs for each matched molecular pair are grouped into three categories based on the shift in t_1/2_: positive (+ ve) shift; negative (−ve) shift; and undetermined (und).

## Discussion

As drug discovery costs continue to rise, it is important to find alternatives to reduce costs and attrition of compounds in the discovery process. Developing and applying in silico tools is one way of reducing cost and optimizing the efficiency of the drug discovery process. Machine learning methods are popularly employed for developing QSAR models that construct relationships between chemical structure and biological properties including ADME. Classification QSAR models relate chemical structures represented as molecular descriptors to a categorical label for the property of interest (i.e. metabolic stability in our case). Several drug candidates have failed due to metabolism or pharmacokinetic issues^50^ and thus it is critical to evaluate metabolic stability very early in drug discovery. While a lot of focus has been geared towards building clearance prediction tools using human liver microsome data^25,51–53^, very little attention has been given to building such tools using rodent data. Clearance prediction models in rodent species, such as rats, are extremely important as a lot of pre-clinical work including efficacy, toxicity and pharmacokinetic evaluations are performed in rats. Using our 24,000 compound Tier I RLM library, we built classification models to predict clearance for test compounds. In addition to helping the chemists rank order compounds and prioritize synthesis, these classification models aid the chemists in identifying potentially unstable compounds that could be optimized for metabolic stability. Using these models to prioritize synthesis will hopefully reduce attrition and get project teams to their lead compounds in fewer iterations. While a couple of RLM clearance models were already reported in literature^6,7^, neither the models nor the data have been made publicly available. We also cannot reveal our entire data set at this time, owing to its proprietary nature, however we do make a subset of data public (AID: 1508591) along with the most predictive models. This is to the best of our knowledge, the first open-access RLM clearance model built using high quality data, generated at a single laboratory.

Based on five-fold cross-validation, GCNN performed better than other machine learning methods employed in this study (Fig. 6). To highlight the importance of our QSAR models, we performed a retrospective analysis on the 10 largest NCATS projects. As expected, the predictive power of the global model increased with increasing number of compounds in the training set. While this global model approach worked well for most projects, some projects performed poorly with this approach. In such cases, a local model strategy could be applied to increase the predictive power compared to the global model. The strengths and weaknesses of global and local QSAR approaches have been previously discussed in literature^54–57^. One study introduced an automated QSAR procedure that involves automatic selection of the most predictive models from a pool of local and global QSAR models^56^. The authors demonstrated that this model selection strategy resulted in a statistically significant improvement compared to regularly updating the global models. Our results are consistent with these findings and we would like to further explore model selection strategies as we generate new data in years to come.

While a lot of rat microsomal stability data exists in the literature, most of the studies focused on addressing specific project related questions. There are few rat liver microsomal stability data sets in literature and only two published studies^6,7^ come close to the scope of the present study. The performance of our best QSAR model is similar if not slightly better than the two aforementioned studies (Table 4). These findings must be cautiously inferred since different data sets were employed in building all these models. Interestingly, one of these studies reported a chronological analysis of the predictive power of models^7^. After building a model using data from one year, they picked 1000 random test set compounds each from four consecutive quarters and evaluated the performance on these subsets. The authors reported a decline in the sensitivity in the last two quarters and understood that the compounds from these subsets were dissimilar to the training set used to build the prediction model. Similarly, we noticed that for some projects the global QSAR model performance declined in year three. We assume that the reason for this decline is the introduction of new chemotypes, previously not covered by the training data.

**Table 4:** Comparison of performance of our best RLM stability model with the literature models.

Metric	Chang et al.^6^	Hu et al.^7^	NCATS RLM (Best Individual Model)	NCATS RLM (Consensus Model)
BACC	0.81	0.77	0.82	0.83
Sensitivity	0.82	0.73	0.86	0.85
Specificity	0.80	0.80	0.77	0.81
Kappa	0.62	0.53	0.64	0.66

One of the previous works that reported global QSAR models also identified good and bad structural features associated with RLM stability from their data set comprising ~ 24,000 compounds^7^. Using naïve Bayesian classifiers, the authors identified fingerprint features that were frequently found in stable and unstable compounds. It must be noted that their t_1/2_ threshold to classify compounds into unstable or stable differs from ours. The fingerprint features identified were ranked using normalized Bayesian probability to identify the top features that are good or bad for stability. Although we did not use the same strategy to identify features within our RLM library, we checked for the presence of substructures proposed by Hu et al*.* within our data set but found only a handful of compounds comprising those features, suggesting that the chemical spaces from both studies may be distinct. Moreover, considering the origin of these features, we were unsure if the fragments identified via such analysis would facilitate synthetically feasible guidance for the chemists to address RLM metabolism liabilities. This led us to investigate alternate ways to analyze the chemical space.

Along these lines, we analyzed the complete data set for the presence of privileged structural motifs that might be overrepresented in stable or unstable compounds by performing RECAP (Retrosynthetic Combinatorial Analysis Procedure) analysis^58^. RECAP generates fragments from molecules based on chemical knowledge. The generated fragments serve as building blocks which make it feasible for the chemists to introduce or replace them during lead optimization. 7000 fragments identified after RECAP analysis were further filtered to retain about 70 scaffolds (top 10 are presented in the supplementary information, Table S6) that contain a minimum of 3 and a maximum of 12 heavy atoms. None of these scaffolds were found to be overrepresented in either compound class. Although some were present more frequently in stable or unstable compounds (15 scaffolds were present at least five times more frequently in either of the classes), the differences did not appear too significant. Furthermore, as Hu et al. suggested, there were high chances that compounds comprising these scaffolds could be analogues sharing a common scaffold, and therefore might not represent a global trend. Thus, we decided against investigating RECAP-based scaffolds/fragments further.

Next, we performed a traditional matched pairs analysis to identify chemical structure transformations that could be useful for chemists to overcome metabolic stability mediated liabilities. As mentioned in the results, a total of 18 transformations were extracted from the training data chosen for MMPA and closely investigated. The following statistics were provided for each of these 18 transforms: number of compound pairs with a positive (+ ve) shift in t_1/2_; number of pairs with a negative (−ve) shift; number of pairs with undetermined (und) shift (when both compounds had t_1/2_ values above 30 min); the average of the t_1/2_ of all compounds on the left and right sides; and the average of the differences in t_1/2_ values between the right and left side compounds. All these transforms led to an average increase in t_1/2_ by at least 10 min in the training data. However, only six of these 18 transforms have at least the same impact on test data. Seven out of the remaining transforms improved t_1/2_ by at least five min, on average. The remaining 5 transforms could only lead to an increase in t_1/2_ by less than five min although none of them on average resulted in a negative shift. Furthermore, each of these transforms represents compounds belonging to multiple projects, indicating the structural/therapeutic diversity of the compounds which in turn supports the hypotheses that some of these rules could be globally applied to improve microsomal stability of small molecules. Considering that CYP450 mediated metabolism is predominant in liver microsomes, we expected to detect transformations that resemble the common mechanisms of metabolism by the CYP enzymes^59^. Several hundreds of compound pairs could be identified that represent N-dealkylation and release of heteroatoms such as halogens. However, the results (data not shown) were mixed since, on average, they did not demonstrate significant changes in t_1/2_. While these represent some of the common mechanisms adapted by the medicinal chemists^60^ to address microsomal stability issues, our results suggest that the protecting effect of such groups (e.g. fluorine) is dependent on the molecular context. Moreover, the complexities involved in generalizing the rules (global versus local) obtained via MMPA and their dependence on molecular context is well acknowledged^61^.

Each in silico model has its limitations and the deficiencies of the experimental methods will be reflected in the model. For instance, it is well recognized that in vitro metabolic stability testing for highly lipophilic and highly insoluble compounds are inclined to errors^6^ and thus, predictive values for such compounds may not be accurate. Apart from the models, we also provide a subset of the RLM stability data set comprised of ~ 2500 compounds, which is by far the largest data set available for RLM stability within the public domain. The models and the data set are available at https://opendata.ncats.nih.gov/adme. The users can also directly predict the RLM stability of new chemical compounds. Alternatively, the models can be downloaded as a self-contained package that can be installed and run locally. The data was also deposited in PubChem (AID: 1508591) and can be directly accessed from the database.

In summary, the work discussed here presents one of the largest in silico analysis of RLM metabolic stability using a curated data set comprising more than 20,000 compounds tested in the same laboratory. The resulting QSAR models could be an invaluable resource to the drug discovery and development community. This is also the first study to the best of our knowledge to release the RLM metabolic stability data for ~ 2500 compounds into the public domain. We explored different modeling strategies and proposed that the choice between global and local approach can be key depending on the data set at hand. Furthermore, the structural insights provided are expected to be useful in overcoming metabolic stability mediated liabilities.

## Supplementary Information

Supplementary Information

## Data Availability

The chemical structures for all compounds in the microsomal stability data set cannot be made publicly available because most of these compounds are part of current active projects at NCATS. However, a subset of the data set, along with the compound structures, is provided along with this study and has also been deposited in PubChem database (AID: 1508591). Furthermore, the best classification models are made available at https://opendata.ncats.nih.gov/adme/.
