# Supplementary material for: Retrospective assessment of rat liver microsomal stability at NCATS: data and QSAR models
Source: Sci Rep. 2020 Nov 26;10:20713. doi: 10.1038/s41598-020-77327-0 (PMC7693334; doi:10.1038/s41598-020-77327-0)
Supplement: Supplementary file 1 — Supplementary Information [file 41598_2020_77327_MOESM1_ESM.pdf]

Supporting Information for

Retrospective Assessment of Rat Liver Microsomal Stability at NCATS: Data and QSAR Models

Vishal B. Siramshetty[1‡], Pranav Shah[1‡], Edward Kerns[1], Kimloan Nguyen1[1,2], Kyeong Ri Yu[1,3], Md Kabir[1,4], Jordan Williams[1], Jorge Neyra[1], Noel Southall[1], Dac-Trung Nguyen[1] & Xin Xu[1*]

1: National Center for Advancing Translational Sciences (NCATS), 9800 Medical Center Drive, Rockville, Maryland 20850, United States

2: NY State Public Health, DOHMH 42-09 28th St, Long Island City, New York 11101, United States

3: School of Medicine, Virginia Commonwealth University, 1201 E Marshall St, Richmond, Virginia 23298, United States

4: The Graduate School of Biomedical Sciences, Icahn School of Medicine at Mount Sinai, 1 Gustave L. Levy Place, New York 10029, United States

‡These authors provided equal contributions.

*Corresponding author

Xin Xu (Email: xin.xu3@nih.gov)

Table S1. Detailed steps involved in preprocessing the RLM data set.

Preprocessing Step	Total Entries
Raw data	23,706
After grouping by internal compound identifiers	21,195
After assigning chemical structures (SMILES)	21,107
After grouping by LyChI (post to structure normalization)	20,385
After omitting compounds with conflicting class labels	20,218
After generation of molecular descriptors	20,216

Table S2. Modeling parameters for the Deep Neural Network model.

Model Parameter	Value
Neurons per layer (5 layers in total)	[2000, 2000, 1000, 700, 1]
Optimizer (learning rate)	Adam (0.0005)
Activation function	ReLu
Number of epochs	30
Batch size	128

Table S3. The inter-assay reproducibility for non-control compounds. Since some of these compounds are part of active projects at NCATS, the compound identifiers are anonymized, and structures are not shown.

Compound	Average t1/2 (min)	S.D	Replicates
Compound 1	17.69	2.73	7
Compound 2	26.58	1.07	4
Compound 3	4.01	0.33	4

Compound 4	3.66	0.69	6
Compound 5	6.85	2.90	4
Compound 6	1.62	0.22	4
Compound 7	27.33	1.96	4
Compound 8	10.33	3.22	9
Compound 9	7.65	0.26	4
Compound 10	19.43	3.25	6
Compound 11	15.50	2.07	6
Compound 12	6.95	0.90	4
Compound 13	2.80	0.83	4
Compound 14	5.19	0.56	4
Compound 15	17.73	7.00	7
Compound 16	21.68	3.92	11
Compound 17	20.88	6.48	4
Compound 18	2.37	0.80	5
Compound 19	1.60	0.07	4
Compound 20	1.32	0.38	5
Compound 21	2.34	0.02	4
Compound 22	1.91	0.39	4
Compound 23	10.75	1.13	10
Compound 24	2.78	0.07	9
Compound 25	16.06	0.60	4
Compound 26	2.77	0.20	4
Compound 27	1.94	0.07	4
Compound 28	20.81	3.63	4
Compound 29	22.03	1.50	4
Compound 30	11.46	2.32	5
Compound 31	11.21	1.20	4
Compound 32	14.30	0.91	5

Compound 33	21.81	4.56	4
Compound 34	15.83	2.97	4

Table S4. Cross-validation results.

Method	Descriptor	Sensitivity	Specificity	Kappa
RF	RDKit	0.86 +/- 0.01	0.75 +/- 0.01	0.61 +/- 0.01
RF	Morgan FP	0.87 +/- 0.01	0.72 +/- 0.01	0.60 +/- 0.01
RF	Avalon FP	0.86 +/- 0.01	0.72 +/- 0.01	0.58 +/- 0.01
DNN	RDKit	0.77 +/- 0.05	0.75 +/- 0.05	0.51 +/- 0.01
DNN	Morgan FP	0.84 +/- 0.02	0.77 +/- 0.03	0.61 +/- 0.02
DNN	Avalon FP	0.83 +/- 0.04	0.76 +/- 0.04	0.59 +/- 0.03
LSTM	SMILES	0.77 +/- 0.04	0.73 +/- 0.04	0.49 +/- 0.01
GCNN	Molecular Graph	0.86 +/- 0.02	0.77 +/- 0.02	0.64 +/- 0.01

Table S5. Time-split validation results.

Dataset	Model	Sensitivity	Specificity	Kappa
2015	RF	0.84	0.55	0.39
	DNN	0.77	0.64	0.41
	LSTM	0.75	0.63	0.37
	GCNN	0.77	0.73	0.50
2016	RF	0.79	0.61	0.40
	DNN	0.76	0.62	0.38
	LSTM	0.59	0.69	0.28
	GCNN	0.73	0.76	0.48
2017	RF	0.80	0.65	0.46
	DNN	0.67	0.69	0.36
	LSTM	0.67	0.65	0.33
	GCNN	0.70	0.74	0.44

2018	RF	0.87	0.54	0.41
	DNN	0.73	0.68	0.41
	LSTM	0.65	0.65	0.30
	GCNN	0.83	0.69	0.52
2019	RF	0.86	0.63	0.50
	DNN	0.85	0.60	0.46
	LSTM	0.79	0.59	0.38
	GCNN	0.82	0.72	0.54

Table S6. Top 10 fragments obtained from the RECAP analysis, sorted by total frequency.

Fragment	Frequency	Frequency (stable)	Frequency (unstable)
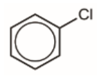	767	486	281
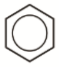	413	185	228
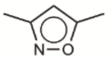	237	63	174
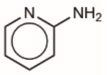	207	132	75
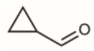	132	49	83
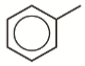	105	8	97
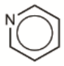	103	29	74
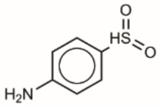	95	18	77

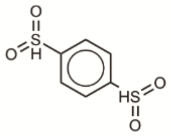	93	1	92
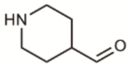	85	12	73
